# Retrieving Precise Three-Dimensional Deformation on the 2014 M6.0 South Napa Earthquake by Joint Inversion of Multi-Sensor SAR

**DOI:** 10.1038/s41598-017-06018-0

**Published:** 2017-07-14

**Authors:** Min-Jeong Jo, Hyung-Sup Jung, Sang-Ho Yun

**Affiliations:** 1 Department of Earth System Sciences, Yonsei University, Seoul, Republic of Korea; 2 Department of Geoinformatics, The University of Seoul, Seoul, Republic of Korea; 3 Jet Propulsion Laboratory, California Institute of Technology, Pasadena, California USA

## Abstract

We reconstructed the three-dimensional (3D) surface displacement field of the 24 August 2014 M6.0 South Napa earthquake using SAR data from the Italian Space Agency’s COSMO-SkyMed and the European Space Agency’s Sentinel-1A satellites. Along-track and cross-track displacements produced with conventional SAR interferometry (InSAR) and multiple-aperture SAR interferometry (MAI) techniques were integrated to retrieve the east, north, and up components of surface deformation. The resulting 3D displacement maps clearly delineated the right-lateral shear motion of the fault rupture with a maximum surface displacement of approximately 45 cm along the fault’s strike, showing the east and north components of the trace particularly clearly. These maps also suggested a better-constrained model for the South Napa earthquake. We determined a strike of approximately 338° and dip of 85° by applying the *Okada* dislocation model considering a single patch with a homogeneous slip motion. Using the distributed slip model obtained by a linear solution, we estimated that a peak slip of approximately 1.7 m occurred around 4 km depth from the surface. 3D modelling using the retrieved 3D maps helps clarify the fault’s nature and thus characterize its behaviour.

## Introduction

**Figure 1:**
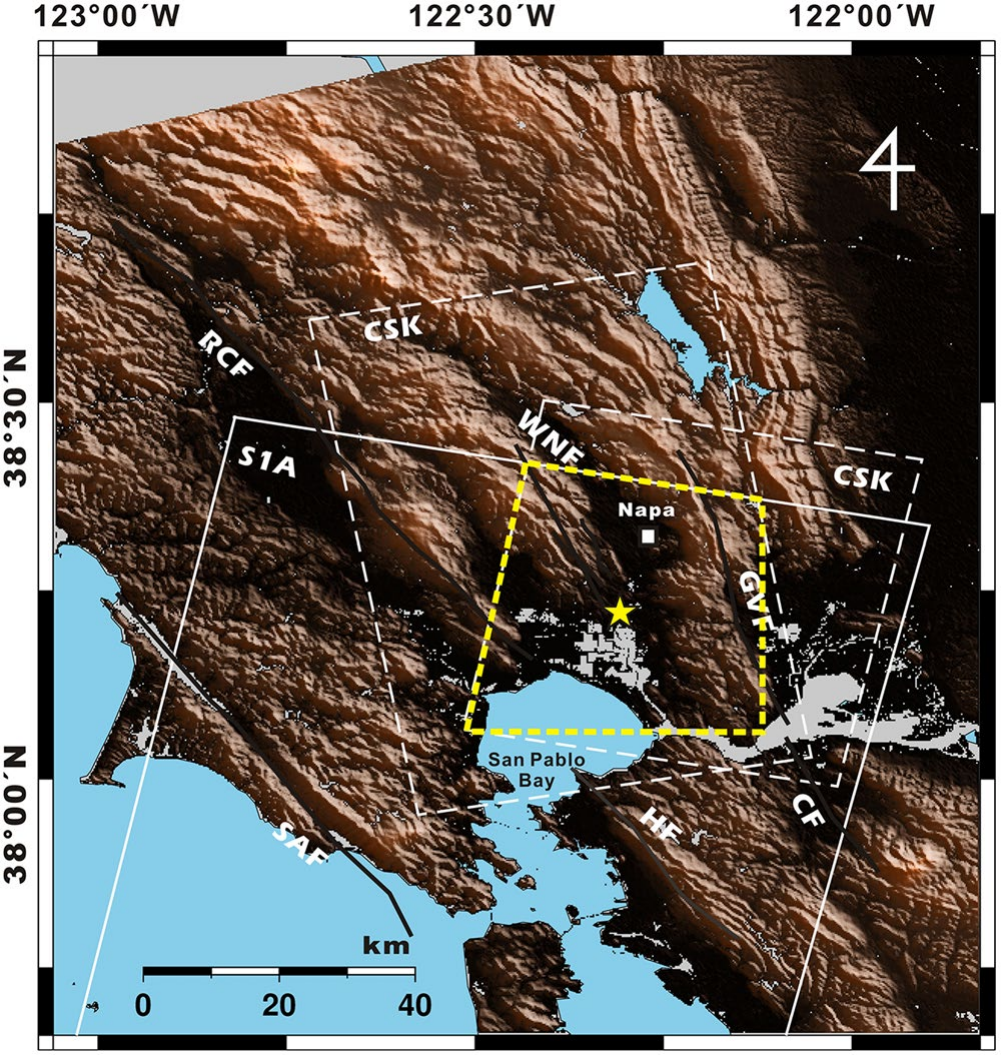
Shaded relief map of the South Napa earthquake region. Thick lines indicate the major faults in the area: SAF, San Andreas Fault; RCF, Rodgers Creek Fault; WNF, West Napa Fault; GVF, Green Valley Fault; CF, Concord Fault; HF, Hayward Fault. The city of Napa and the epicentre are shown as a small square and star, respectively. White rectangles with dashed and solid lines show the frames of COSMO-SkyMed (CSK) and Sentinel-1A (S1A) SAR images, respectively. 3D displacements were retrieved for the areas overlapped by all data frames (yellow dashed line). The map was generated by using the generic mapping tools (GMT) software 5.3.2 version (http://gmt.soest.hawaii.edu/projects/gmt/wiki/Download).

The M6.0 South Napa earthquake occurred in the vicinity of Napa, California on 24 August, 2014. The 12-km-long surface fault rupture occurred on the West Napa Fault with a dextral strike-slip shear sense (Fig. 1). In order to quantitatively analyze the surface deformation caused by this earthquake, we carried out a geodetic analysis using interferometric synthetic aperture radar (InSAR).

Interferometric SAR (InSAR) is a powerful technique for measuring ground surface deformation, with a swath width of at least tens of kilometres and a resolution on the order of tens of metres. InSAR measurements are especially useful for the analysis of large-scale earthquakes, which can occur over hundreds of kilometres, because it can quickly examine the scale and scope of damage scale. InSAR observations can also generate coseismic dislocation models, which provide information regarding the maximum slip and source geometries^1–3^. Such studies require the use of SAR amplitude-based sub-pixel offset maps or GPS measurements to supplement the components missing from InSAR measurements^4–6^. Although InSAR has become an essential technique for measuring surface deformation, it has insufficient resolution power relative to the north component of three-dimensional (3D) measurements for precise observations.

The measurement of 3D surface displacement has been remarkably improved by the development of multiple-aperture SAR interferometry (MAI)^7, 8^. Some studies have used a split spectrum in the azimuth domain for image co-registration, a method which has developed into the MAI technique for measuring surface displacement in the along-track direction^9, 10^. The integration of InSAR and MAI measurements from different SAR geometries has been used to produce improved 3D displacement maps with a precision of a few centimetres^11, 12^.

In this study, we reconstructed the precise 3D displacements of the 2014 South Napa earthquake’s surface deformation using the joint inversion of InSAR and MAI measurements from two spaceborne platforms. Multiple interferometric pairs for coseismic deformation were collected from the Sentinel-1A (S1A) satellite, which carries a C-band SAR sensor for the purpose of Earth observation and natural disaster mapping. The South Napa earthquake is the first geological event covered by this mission since the satellite’s launch on 3 April, 2014. The S1A mission can use the Terrain Observation by Progressive Scans (TOPSAR) acquisition method to produce high-quality wide-swath images; we used a pair of stripmap mode images acquired in this manner for observing the earthquake zone.

The other satellite data source used in this study, COSMO-SkyMed (CSK), is an X-band SAR system comprised of four satellites in the same orbital plane with an uneven temporal separation. These satellites captured the coseismic deformation of the South Napa earthquake with high temporal resolution. In a previous study, modelling of the South Napa earthquake was constrained by both InSAR and GPS measurements and so slip distributions were only estimated^13^. However, a precise 3D displacement map from the MAI-based method was not computed for the event. In the current study, we retrieved 3D components of surface deformation by integrating three line-of-sight (LOS) displacement maps with two along-track displacement maps from multi-sensor SAR acquisitions. We expected to produce a better-constrained slip model from the 3D displacement maps by reducing the uncertainty of the model parameter estimation.

## South Napa Earthquake

The 2014 South Napa earthquake that occurred on 24 August, 2014 at 10:20 UTC was the largest earthquake in the San Francisco Bay area since the Loma Prieta earthquake in 1989. The shock occurred as a consequence of activity within the San Andreas Fault system which forms the plate boundary between the Pacific and North American tectonic plates in this area. According to the U.S. Geologic Survey (USGS), the earthquake’s epicentre was located near the West Napa Fault (122° 18′36″ W, 38°13′12″ N, 11.3 km depth) between two major fault systems, the Hayward-Rodgers Creek Fault system and the Concord-Green Valley Fault system, both of which are characterized by dextral strike-slip motion (Fig. 1). The West Napa Fault is 35 kilometres long, trends approximately north-northwest (NNW), and has a relatively minor strike-slip motion caused by activities within the surrounding major fault systems; it is likely that the South Napa earthquake occurred on this fault^13–15^.

## Methods

In order to retrieve the 3D components of surface deformation for the 2014 South Napa earthquake, we first measured the LOS and along-track displacement using InSAR and MAI for three interferometric pairs acquired from different geometries (Table 1). Single pairs of ascending and descending images were collected from the CSK satellite along with a single pair of descending images from the satellite S1A. The descending CSK pair covers potential postseismic deformation over 3 days, while the ascending CSK and descending S1A pairs cover 10 and 7 days, respectively. We also collected additional CSK data in a descending orbit in order to match the repeat pass timing of the S1A pair, but did not use the additional data for analysis as we found insignificant deformation signals from the 3-day postseismic interferogram; the pair of images caused an increase in unwanted signal noise rather than meaningfully coherent signals. Thus, we only used the initial three coseismic pairs to retrieve the 3D components of surface deformation by combining the InSAR and MAI measurements from all pairs.

**Table 1:** Characteristics of interferometric pairs used in this study.

Sensor	Mode	Master	Slave	*f*_*DC,f*_^1)^ (Hz)	*f*_*DC*_,_*c*_^1)^ (Hz)	*f*_*DC*_,_*b*_^1)^ (Hz)	*Δf*_*D*_,_*S*_^1)^ (Hz)	*B*_⊥_^2^ (m)	*ΔB*_⊥_^2^ (m)
CSK	DSC	07/26/2014	08/27/2014	1177.6	540.7	−96.13	1271.0	−134.7	0.0002
	ASC	06/19/2014	09/03/2014	−184.3	−820.2	−1456.0	680.7	162.9	0.0003
S1A	DSC	08/07/2014	08/31/2014	415.3	1.89	−377.3	766.6	−2.0	0.000

^1)^ *f*_*DC*_,_*f*_, *f*_*DC*_,_*c*_, and *f*_*DC*_,_*b*_ denote the forward, average, and backward Doppler Centroids, respectively, and *Δf*_*D*_,_*S*_ is the sub-aperture processing bandwidth.

^2)^B_⊥_ is the perpendicular baseline of the forward-looking interferogram and *ΔB*_⊥_ is the perpendicular baseline difference.

The major data-processing difference between CSK and S1A SAR data is the generation of the sub-aperture single-look complex (SLC) in the MAI processing scheme. We initiated MAI processing by using raw SAR signals for both descending and ascending CSK data. Four SLC images, composed of both forward- and backward-looking SLC images from the master and slave SAR data, were generated by controlling the Doppler Centroids and processing bandwidth (*PBW*) for sub-aperture images according to the MAI processor^8^. On the other hand, we obtained SLC images from the S1A SAR system because they were the only available data format from the data distribution centre. The MAI processing for the SLC data includes azimuth common-band filtering between the master and slave SLC images and beam-splitting for the purpose of dividing forward- and backward-looking spectrums.

After separating out two forward-looking and two backward-looking SLC images from a single InSAR pair, both forward- and backward-looking interferograms were independently generated. MAI interferograms were then computed using complex conjugate multiplication between sub-aperture interferograms for each of the CSK and S1A SAR data, as described by Jung *et al*.^8^. Along-track displacements (Δ*x*) using the MAI method can be defined as follows^7^. For the CSK system beginning with raw signals,

1 $${\rm{\Delta }}x=-\frac{l}{4\pi n}{\phi }_{MAI},$$

where *l* is the effective antenna length (5.7 m for CSK), *ϕ*_*MAI*_ is the MAI phase, and *n* is the fraction of the full-aperture beam width, which was set to a value of 0.5 in this study.

Meanwhile, the effective antenna length needs to be compensated for in MAI processing starting from SLC images because the processing bandwidth of the azimuth spectra is pre-determined during the SLC generation. Thus, the azimuth antenna length ${\hat{l}}_{az}$ is recalculated as follows:

2 $${\hat{l}}_{az}=\frac{{2}{{V}}_{{g}}}{PB{W}_{{\rm{e}}}}$$

where *v*_*g*_ is satellite velocity along the Earth’s surface (approximately 12% less than the orbital velocity *v*_*s*_^16^) and *PBW*_*e*_ is the effective processing bandwidth calculated by subtracting the Doppler centroid difference between master and slave data from the processing bandwidth *PBW*.

For the S1A data, we recalculated the original antenna length of 12 m to 9.85 m; using this compensated antenna length, the along-track displacement (Δ*x*) for S1A descending data is calculated as follows:

3 $${\rm{\Delta }}x{\boldsymbol{=}}-\frac{{\hat{l}}_{az}}{4\pi n}{\varphi }_{MAI}$$

During the processing for both InSAR and MAI, we applied sixteen-looks in the range and azimuth directions for CSK and S1A data in order to reduce phase noises in the interferograms. After the data reduction, the pixel spacings within the final InSAR and MAI interferograms were approximately 40 m and 60 m for the CSK and S1A images, respectively. We removed topographic phase contributions using a simulated interferogram derived from a one arc-second shuttle radar topographic mission (SRTM) digital elevation model (DEM)^17^.

We reconstructed the 3D displacement map by integrating three LOS and two along-track displacement maps measured from the CSK and S1A data^4, 5, 18^. All measurements were jointly inverted to reconstruct the east (*u*_*e*_), north (*u*_*n*_), and up (*u*_*u*_) components of the earthquake’s surface deformation. The satellites’ look vectors for InSAR (*d*_*los*_) and MAI (*d*_*MAI*_) measurements used in this study are defined as follows:

4 $$[{u}_{n}\,\sin \,\phi -{u}_{e}\,\cos \,\phi ]\,\sin \,\theta +{u}_{u}\,\cos \,\theta ={d}_{los}$$

5 $${u}_{n}\,\cos \,\phi +{u}_{e}\,\sin \,\phi ={d}_{MAI}$$

Track angles (*φ*) of −9.82° and −169.1° and look angles (*θ*) of 40.02° and 29.5° were used for ascending and descending CSK data sets, respectively, and a *φ* of −168.27° and *θ* of 23.2° were used for the S1A descending data set. We then produced the earthquake model using the 3D displacement maps, which were generated by GAMMA software (http://www.gamma-rs.ch/no_cache/software.html) based on the method proposed and improved by refs 8 and 19, and displayed these using GMT software.

## Results

### Measurements of LOS and along-track displacements

To measure the coseismic surface deformation resulting from the earthquake, we generated InSAR and MAI interferograms from different geometries, constrained by the CSK and S1A data sets (Table 1). The perpendicular baseline of the CSK ascending pair was within an acceptable range, but the MAI interferogram it generated (which we intended to use for 3D component retrieval) had a very low interferometric coherence and so was not used. Despite the small difference in the perpendicular baseline between the CSK ascending and descending pair, their interferometric qualities were substantially different (Fig. S1). While clear and strong signals were identified from the S1A and CSK descending interferograms, relatively weak deformation signals with significant noise were observed from the CSK ascending pair. This primarily resulted from the smaller processing bandwidth of the CSK ascending pair, approximately half that of the descending pair, which seems to have induced a severe decorrelation of the interferogram. Nevertheless, the InSAR interferogram from the CSK ascending pair was adopted as an input for retrieving the 3D displacements, since we needed at least one InSAR observation from an ascending orbit. The 3D displacement maps were subsequently computed by integrating three InSAR and two MAI measurements. The original coverage of each InSAR and MAI interferogram are shown in Fig. S2, but only the overlapped areas have been restored three-dimensionally.

**Figure 2:**
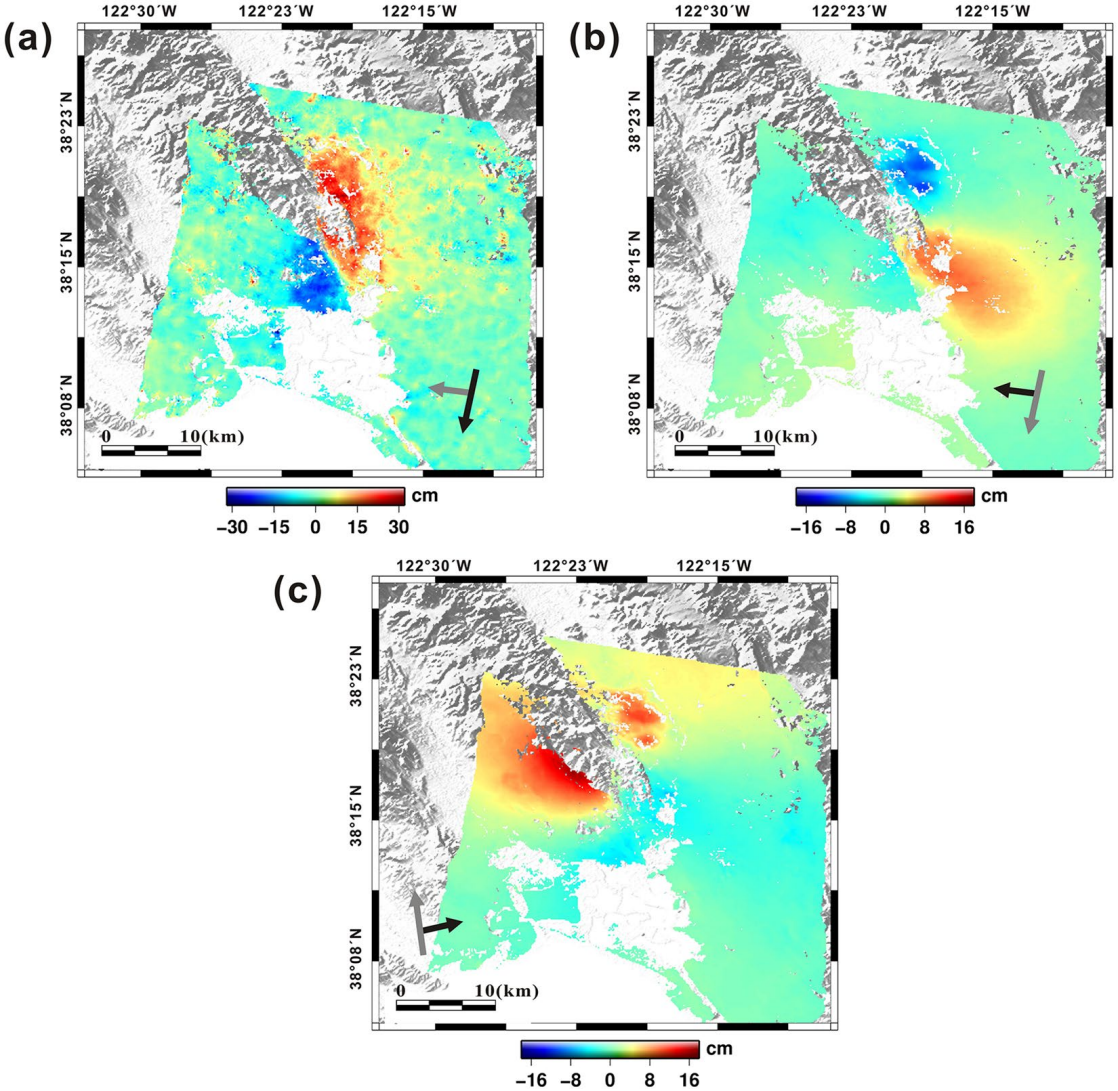
Maps of (**a**) along-track and (**b**) cross-track (LOS) displacements from CSK descending data between 26/07/2014 and 27/08/2014, and (**c**) cross-track displacements from CSK ascending data between 19/06/2014 and 03/09/2014. The displacement maps were generated by the GMT software 5.3.2 version (http://gmt.soest.hawaii.edu/projects/gmt/wiki/Download).

**Figure 3:**
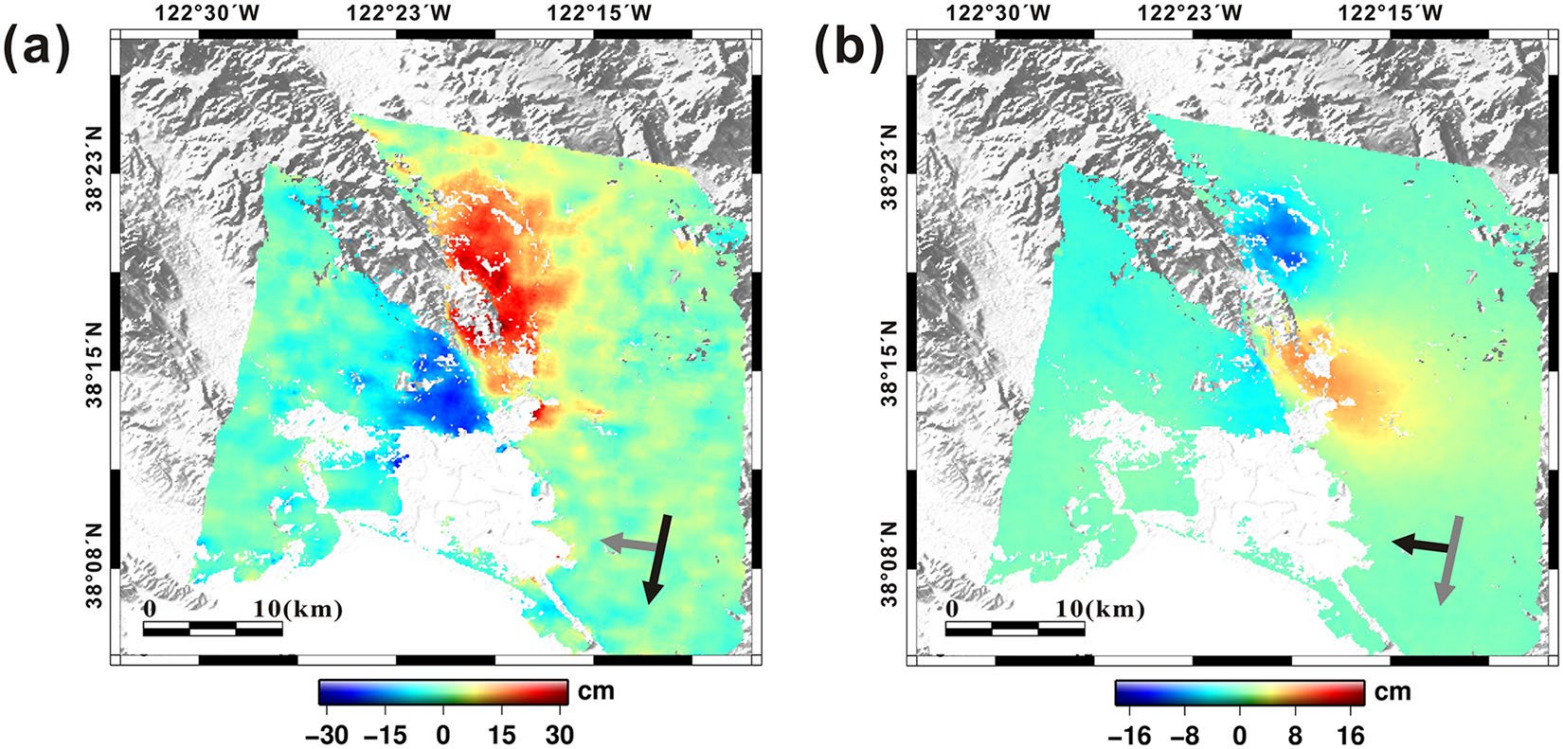
Maps of (**a**) along-track and (**b**) cross-track displacements from S1A descending data between 07/08/2014 and 31/08/2014. The displacement maps were produced by the GMT software 5.3.2 version (http://gmt.soest.hawaii.edu/projects/gmt/wiki/Download).

Figure 2a and b show the along-track and cross-track (LOS) displacements measured from the CSK descending data sets. We masked the de-correlated regions out from the interferograms and removed most pixels within the vicinity of the rupture due to rapid phase gradients or ground surface disturbances. Ground movements related to the earthquake occurred over a period of one month due to the mainshock and aftershock sequences^14^. The MAI interferogram generated from the CSK descending data clearly show the rupture line of the earthquake defined by opposite-directional displacements on the southern edge of the fault zone (Fig. 2a). Positive displacement (roughly southward movement) and negative displacement (roughly northward movement) of the ground are shown along the right- and left-hand side of the fault, respectively, clearly illustrating the dextral strike-slip shear sense of the fault during the South Napa earthquake. The MAI interferogram generated from the descending S1A data produced a very similar result to those of the CSK data (Fig. 3a), also showing opposite directional movements on the left- and right-hand sides of the fault with an obvious dextral strike-slip motion. The measured maximum surface displacement in the along-track direction was ~43 cm using both the CSK and S1A descending data, which can be “back-projected” to the fault strike (161°) as ~45 cm of the surface slip.

The LOS displacements obtained from both the descending and ascending CSK data demonstrated nearly opposite measurement signs (Fig. 2b and c), indicating that surface deformation in the vicinity of the South Napa earthquake was dominantly induced by horizontal movements. Clear features of the surface rupture are especially visible within the LOS and along-track displacement maps from the descending data. Although the rupture was detected equally by both the CSK and S1A descending pairs, the latter’s measurements were slightly smaller, similar to the results obtained from the along-track displacements. On the other hand, it is difficult to observe the surface rupture from the LOS displacement map of the CSK ascending pair because the observing direction of the InSAR measurements in this case is not sensitive to the dominant displacement direction of the earthquake. The measured maximum LOS displacement recorded by the ascending data was approximately 13 cm.

### 3D deformation retrieval

**Figure 4:**
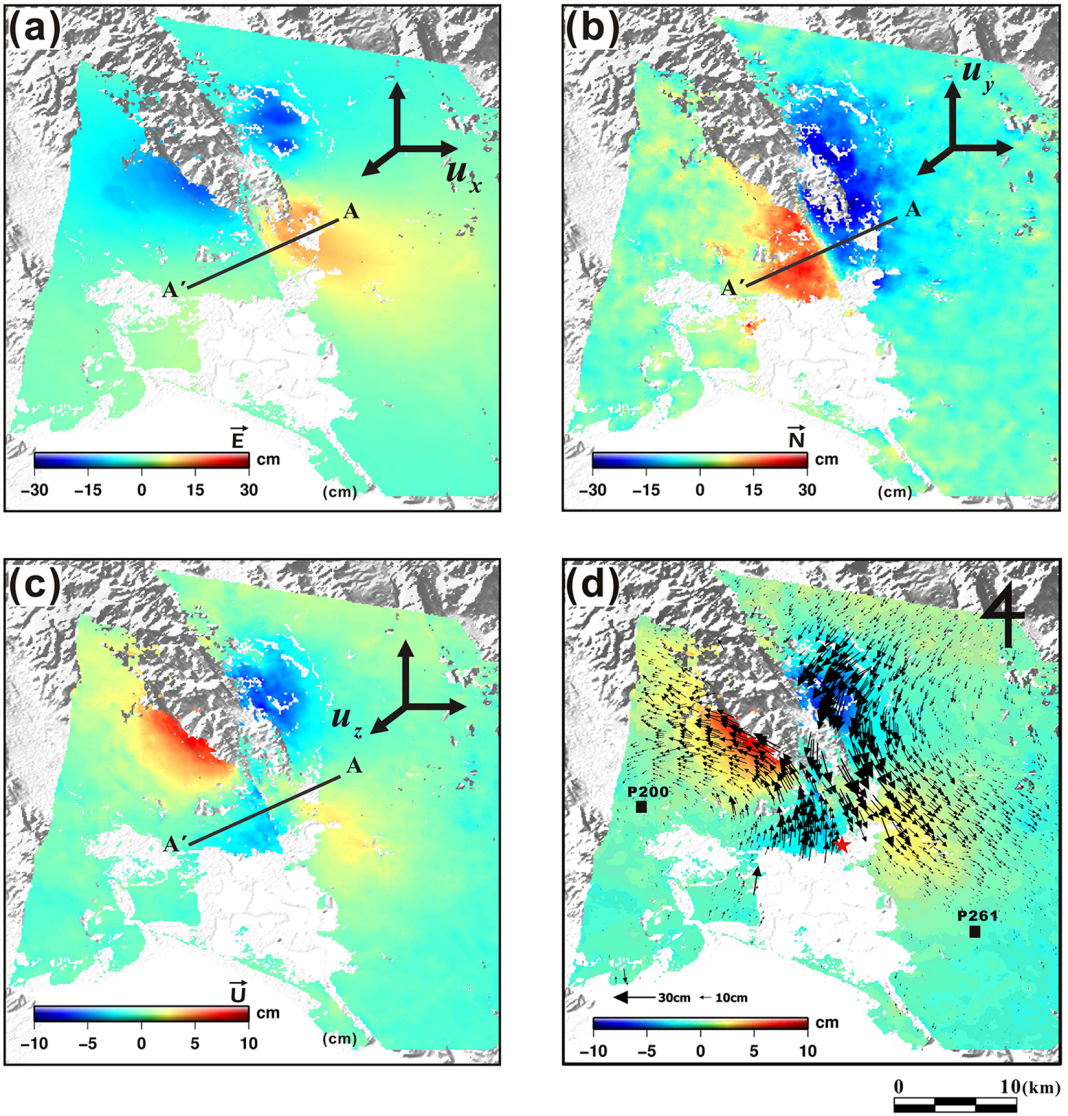
3D surface displacement maps derived by combining multiple InSAR and MAI displacements measured from CSK and S1A data: (**a**) east, (**b**) north, and (**c**) up components of displacement. (**d**) 3D displacement vector field with arrows showing horizontal movement and colours showing vertical movement from the earthquake. Black small squares and red star represent the locations of GPS stations and the epicentre, respectively. Profile A-A’ is shown in more detail in Fig. 5. The maps were generated by using the GMT software 5.3.2 version (http://gmt.soest.hawaii.edu/projects/gmt/wiki/Download).

We reconstructed 3D displacement maps by integrating LOS with along-track displacements measured from multi-sensor SAR data sets. The retrieval of the east (*u*_*e*_), north (*u*_*n*_), and up (*u*_*u*_) components of the surface deformation followed methods described in previous studies^4, 5, 18^ (Fig. 4).

The east component map (Fig. 4a) shows somewhat similar displacement patterns with the LOS deformation measured by the CSK (Fig. 2b) and S1A (Fig. 3b) descending pairs, because horizontal motion was dominant during the earthquake. The north component map (Fig. 4b), which was measured by the MAI method and is mainly dependent on the along-track deformation, clearly showed the characteristics of the earthquake and confirms the dextral strike-slip shear sense of the motion. The maximum relative surface displacement was approximately 42.5 cm toward the south, which can be interpreted as a relative displacement of approximately 45 cm in the direction of the fault strike. This result is similar to the assessment provided by the USGS^14^. The earthquake demonstrated dominantly horizontal motion along a dextral strike-slip fault, but it also encompassed weak vertical movement (Fig. 4c); the measured maximum vertical displacement was approximately 9 cm.

The displacement vectors shown in Fig. 4d were computed by combining the east and north components of the 3D measurements. The heading and length of the figure’s arrows indicate the direction and magnitude of the horizontal displacements, respectively. These vectors display a typical butterfly-shaped displacement pattern along the strike-slip fault within the earthquake region^4, 20, 21^. However, some differences between the seismological observations and InSAR measurements are visible. For example, the epicentre marked in Fig. 4d is not located in the centre of the surface deformation map as derived from satellite data.

**Figure 5:**
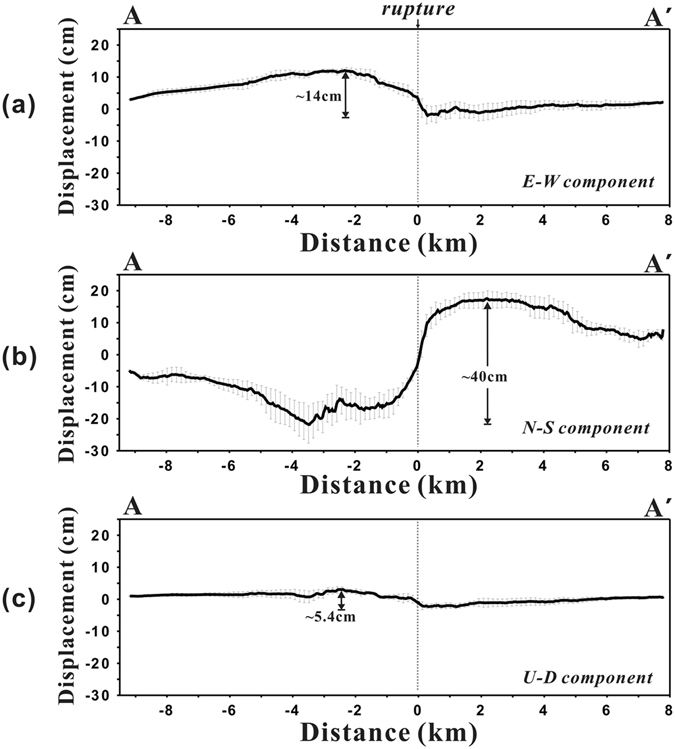
Surface displacements of (**a**) east, (**b**) north, and (**c**) up components along profile A-A’ across the surface rupture shown in Fig. 4.

Figure 5 shows the surface displacements of the east, north, and up components, along the profile A-A’ in Fig. 4. The bold lines representing the displacements for each component were calculated by averaging approximately 150 pixels across the profile, and the error bars indicate the standard deviations. Additionally, the exact position of the fault trace can be detected from the profile where it passes through the surface rupture. Surface displacements of the east and north components are almost zero immediately next to the rupture location. We also compared the 3D displacements with GPS measurements obtained from two stations located within the boundaries of this study (Fig. 4d). Since the distance of either station from the Napa fault was relatively large, the amount of observed displacement was small, roughly 1–2 cm on the north and east components (Fig. S3). We have thus confirmed that the east and north displacements of the retrieved 3D maps show similar displacements as those reported by the GPS stations.

### Modelling

We used a finite dislocation model^22, 23^ with isotropic, homogeneous, and elastic half-space conditions to estimate the fault’s geometry, including length, width, depth, strike, dip, latitude/longitude coordinates, and slip along the strike and dip directions. We assumed a Poisson’s ratio of 0.25 for the modelling. According to the convention of the Okada model, a dip of 90° demonstrates a vertical plane and dips ranging from zero to 90° show a southward sloping plane. In this study, we first determined the fault geometry and then estimated the slip distributions on the fault plane for the multiple patches.

Since the general northwest-southeast trend of the West Napa Fault is favourable to the observation direction of the SAR satellite, particularly for a descending orbit, a few studies have been able to obtain meaningful results using InSAR alone or InSAR combined with GPS measurements^13, 14, 24^. We derived several models under various input conditions and determined the optimal model using 3D measurements. During the model parameter estimation, no weightings were considered for the 3D components, although though the north component was mostly constrained by MAI measurements (which have larger uncertainties than InSAR in general. The InSAR measurements, which almost constrained the east and up components, have undesirable signal noise from atmospheric effects, and we cannot be sure that this noise was sufficiently removed by using only a few InSAR pairs.

**Figure 6:**
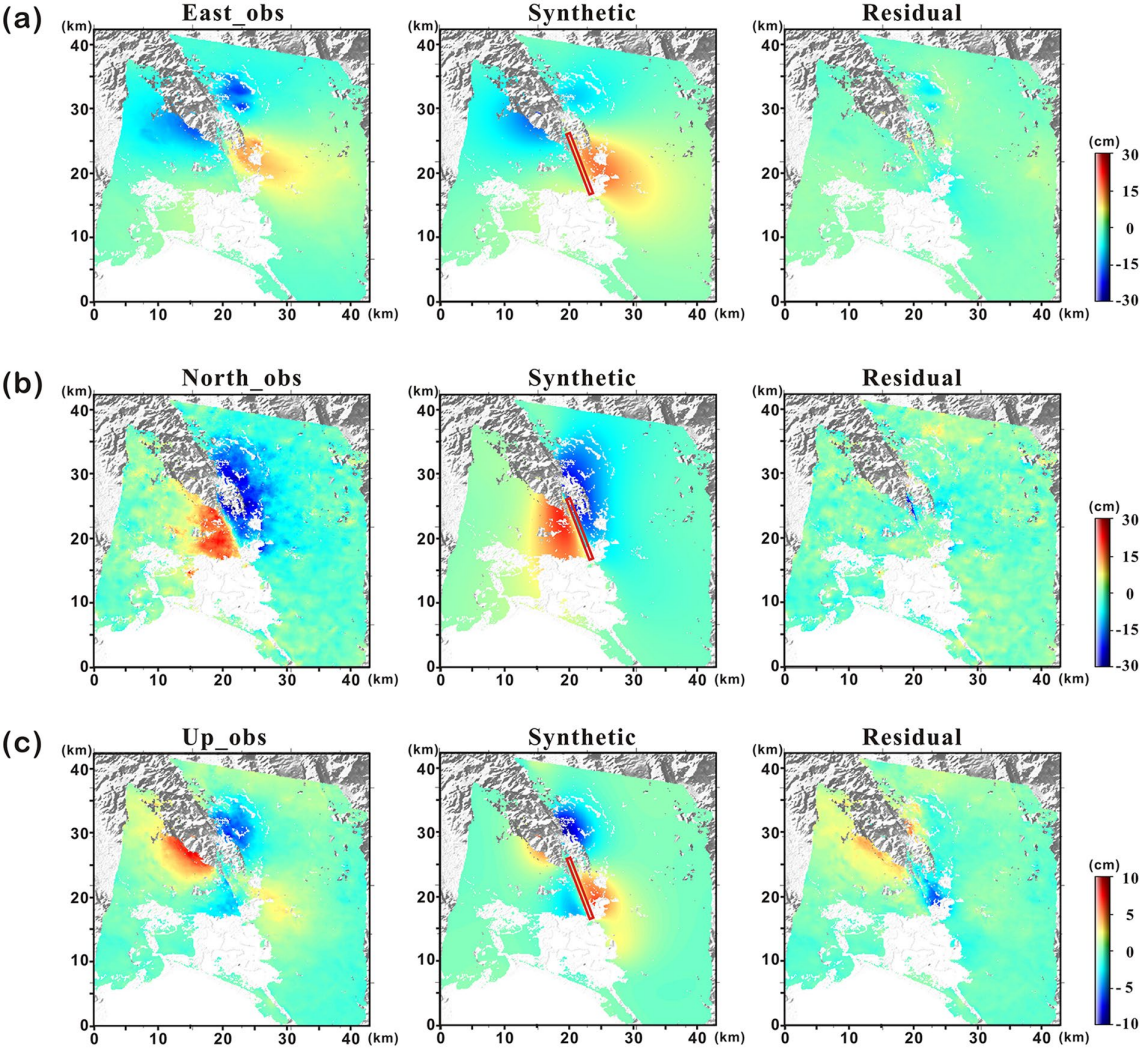
Inversion results of the dislocation model for the South Napa earthquake using 3D displacement maps: observed (left), synthetic (centre), and residual (right) displacements of the (**a**) east, (**b**) north, and (**c**) up components of surface deformation. The displacement maps were produced by using the GMT software 5.3.2 version (http://gmt.soest.hawaii.edu/projects/gmt/wiki/Download).

Figure 6 shows the inversion results from the dislocation model, constrained by the 3D displacement maps, in which the east, north, and up components of surface deformation were used to estimate the geometry of the fault and the amount of fault slip. Relatively large residual signals were identified from the inversion results of the up component, while the east and north components were well-modelled with less of a residual signal. The right side of the rupture, especially in the southern parts of Napa, was not modelled well (Fig. 6a). This area is an urban zone developed on an alluvial fan deposit, and numerous buildings received severe earthquake damage^25^. Some mismatched patterns at this site were induced by the complex deformation of these regions. A combination of urban structures and unconsolidated sediments in this area produced atypical deformation patterns for a strike-slip fault, while the left-hand side of the rupture showed relatively typical deformation patterns. The total root-mean-square (RMS) ratio between the residuals and observations of the 3D modelled result was approximately 0.51, slightly smaller than the modelled result using the three InSAR and two MAI measurements directly. Although unwanted biases can be introduced to the retrieved 3D maps, these enabled us to produce a better slip model with lower residuals, because each InSAR and MAI measurement also contains the signal noise and errors.

Table 2 summarizes the best-fit model parameters estimated from the 3D measurements by a Monte-Carlo simulation of ten thousand iterations using a single patch with uniform slip. The rupture length determined here (10.48 km) is slightly shorter than that observed in the deformation map (12.5 km). We also determined the fault geometry, wherein the strike of the fault via the 3D modeled results is approximately 338.42° with a nearly vertical plane of 84.75° dipping toward the east. As the slip was mostly occurred along the fault strike, a rake and slip was approximately −179.5° and 1.38 m for the uniform dislocation model. There is also some disagreement concerning the strike and dip directions of the fault among numerous related studies^13, 24, 26, 27^. The probable focal planes are roughly 340° (east-dipping) or 160° (west-dipping). We consequently derived a west-dipping fault model considering a strike of 160° and determined that a strike/dip of 159.48°/76° best fit the 3D displacement data. Figure S4 shows the inversion results for this west-dipping fault model, which also fit well with the 3D inputs, although the residuals were a little greater than those in the east-dipping model. The RMS ratio between the residuals and observations of the west-dipping model was approximately 0.63.

**Table 2:** Best-fit dislocation model parameters estimated from 3D displacement maps.

	*Model Parameters*								
Input data	Length (km)	Width (km)	Depth (km)	Strike (Deg)	Dip (Deg)	X (km)	Y (km)	Strike-Slip (m)	Dip-Slip (m)
**3D**	10.4765 ± 1.197	4.5138 ± 0.705	5.4974 ± 1.234	338.4242 ± 6.732	84.7503 ± 4.120	23.7942 ± 1.129	21.8000 ± 1.205	−1.385 ± 0.159	0.010 ± 0.072

*One-sigma uncertainties with optimum model parameters.

**The ground coordinates at the origins of the X and Y axes are −122.545833 and 38.460556 deg, respectively.

**Figure 7:**
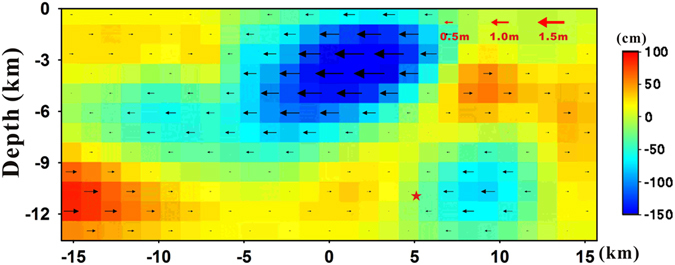
Slip distributions along the fault plane during the South Napa earthquake derived from InSAR-based 3D modelling. Red star represents the hypocentre of the South Napa earthquake.

After determining the model parameters for the geometry of the fault using a simple dislocation model, we solved the distributed fault model for a larger fault than determined in the prior stage using a least-square inversion based on Green’s function while assuming nearly pure strike-slip fault. A Laplacian smoothing operator was also applied during the inversion. As seen from the multiple slip patches in Fig. 7, the peak slip was estimated at ~4 km depth and ~1.7 m along the fault strike. As other studies reported that peak slip occurred between 2.5 km and 7 km depth, this estimate fell within expectations^13, 14, 24, 27^. The distributed slip model also showed that slip occurred primarily between 0 and 6 km depth.

The InSAR-derived fault characteristics from this study have some discrepancies when compared with the seismological solution. While both InSAR and seismic analysis demonstrated the dextral strike-slip motion of the fault, the origin and the geometry of the fault was slightly different between the two. As shown in Fig. 7, the origin reported from other studies is about 11 km deep^14, 27^, more than twice as deep as those of the peak slip derived from the 3D modelled results. In addition, the Northern California Seismic System (NCSS) reported that the most preferred geometry of the fault plane from the moment tensor solution was a strike/dip/rake of 155°/82°/172°. The USGS National Earthquake Information Center (NEIC) provided a similar moment tensor solution of 157° and 77° for the strike and the dip angles. These fault plane solutions are a better match with the modelled results for the west-dipping fault in this study. Only the Lamont-Doherty Earth Observatory Global CMT (GCMT) reported an east-dipping fault plane from the moment tensor. However, the focal mechanism derived from the NCSS provided nodal planes characterizing an east-dipping fault with a strike/dip of 345°/85°. The strike of this fault plane is a little closer to the north-south direction than our modelled results, but dip angle is nearly the same. Thus, we confirmed that the focal mechanism solution was well-matched with the fault model derived from 3D modelling. ﻿Moreover, 3D modelling enabled us to obtain the high resolution focal depth of the earthquake compared to the seismic analysis.

These results are within a reasonable range as defined by other satellite-based studies, but our analysis is unique due to the reconstruction of better-constrained 3D surface movements and the derivation of the fault’s optimal focal plane. These results support existing seismological interpretations and contribute to a better understanding of the West Napa Fault’s behaviour.

## Conclusions

We reconstructed the 3D displacements of surface deformation for the South Napa earthquake by combining MAI and InSAR interferograms from coseismic pairs of X-band COSMO-SkyMed SAR data (from ascending and descending orbits) in addition to C-band Sentinel-1A SAR data (from a descending orbit). The east, north, and up components of surface deformation were retrieved by integration of three InSAR and two MAI measurements. The 3D displacement maps clearly identified dextral shear with a maximum surface slip of approximately 45 cm and weak vertical movement of up to 7 cm. The east, north, and up components of surface deformation were jointly modelled using a finite dislocation model considering a single patch and homogeneous slip of the fault. We then carried out a Monte-Carlo simulation of ten thousand iterations to determine the best-fitting model parameters, particularly with regard to the fault’s strike and dip. Although many previous studies have presented arguments regarding the fault characteristics, this study constrained the geometry of the east-dipping fault possessing a strike of approximately 338° and a dip of 84.7° (according to the 3D displacement maps). This result matches seismological data produced by the focal mechanism and supports a clear interpretation of the fault’s characteristics. We also estimated the slip distribution along the fault plane using the least square inversion method, finding that slip mostly occurred above ~6 km depth with peak slip of ~1.7 m at ~4 km depth along the fault strike. In conclusion, 3D modelling plays a crucial role in determining geologic models for faulting events by reducing uncertainties in model parameter estimations and should be increasingly used to gain a better understanding of geologic processes, particularly for areas demonstrating complex surface deformation.

## Electronic supplementary material

Supporting Information
