# Supplementary material for: Retrieving Precise Three-Dimensional Deformation on the 2014 M6.0 South Napa Earthquake by Joint Inversion of Multi-Sensor SAR
Source: Sci Rep. 2017 Jul 14;7:5485. doi: 10.1038/s41598-017-06018-0 (PMC5511219; doi:10.1038/s41598-017-06018-0)
Supplement: Supplementary file 1 — Supporting Information [file 41598_2017_6018_MOESM1_ESM.pdf]

Supporting Information

Retrieving Precise Three-Dimensional Deformation on the 2014 M6.0 South Napa Earthquake by Joint Inversion of Multi-Sensor SAR

Min-Jeong Jo[1)]

Hyung-Sup Jung[2)*]

Sang-Ho Yun[3)]

[1)] Department of Earth System Sciences, Yonsei University, Republic of Korea

[2)] Department of Geoinformatics, The University of Seoul, Republic of Korea

[3)] Jet Propulsion Laboratory, California Institute of Technology, United States

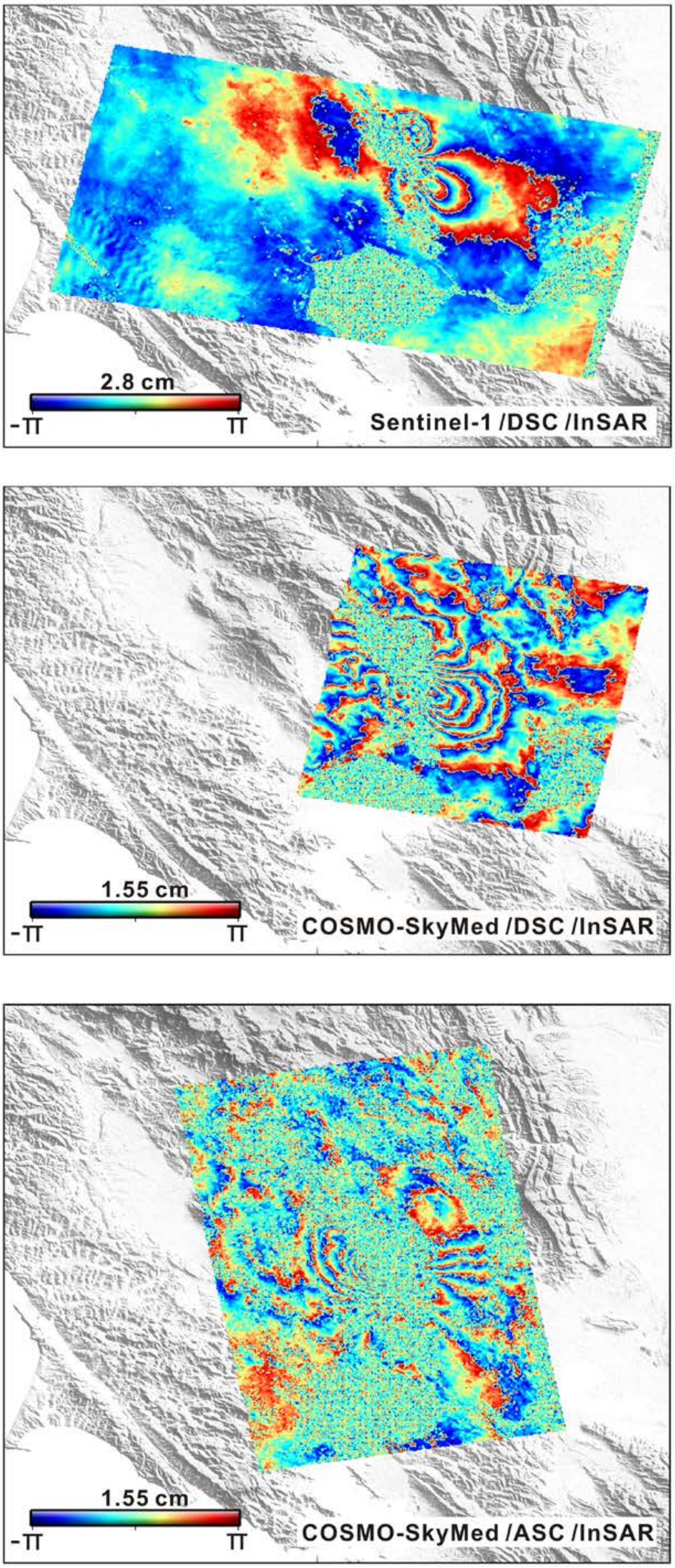

Fig. S1. The wrapped interferograms measured from Sentinel-1 descending and COSMO-SkyMed descending and ascending images. The maps were generated by using the generic mapping tools (GMT) software 5.3.2 version (http://gmt.soest.hawaii.edu/projects/gmt/wiki/Download).

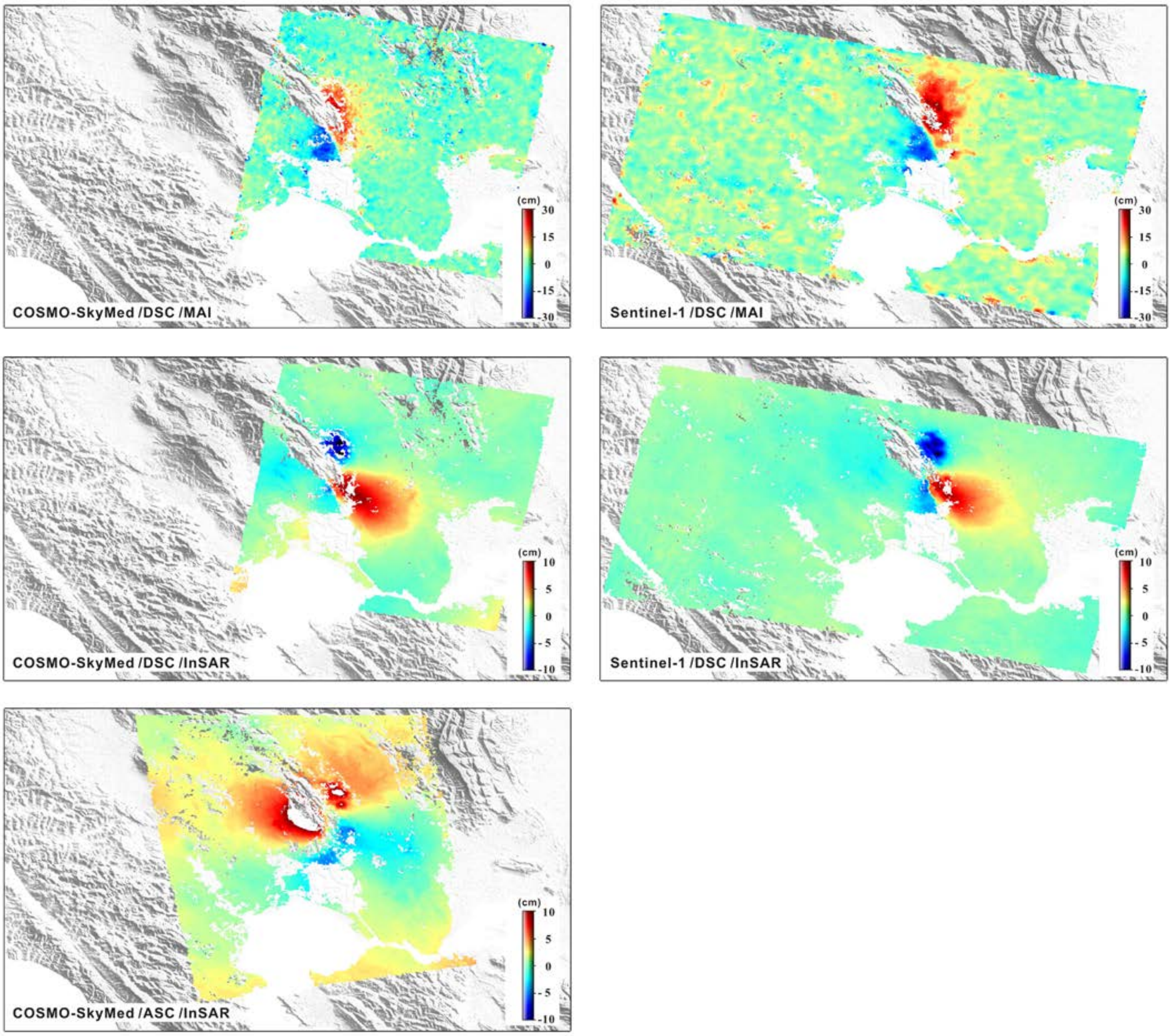

Fig. S2. The original coverage of each LOS and along-track displacements map. The maps were produced by using the GMT software 5.3.2 version (http://gmt.soest.hawaii.edu/projects/gmt/wiki/Download).

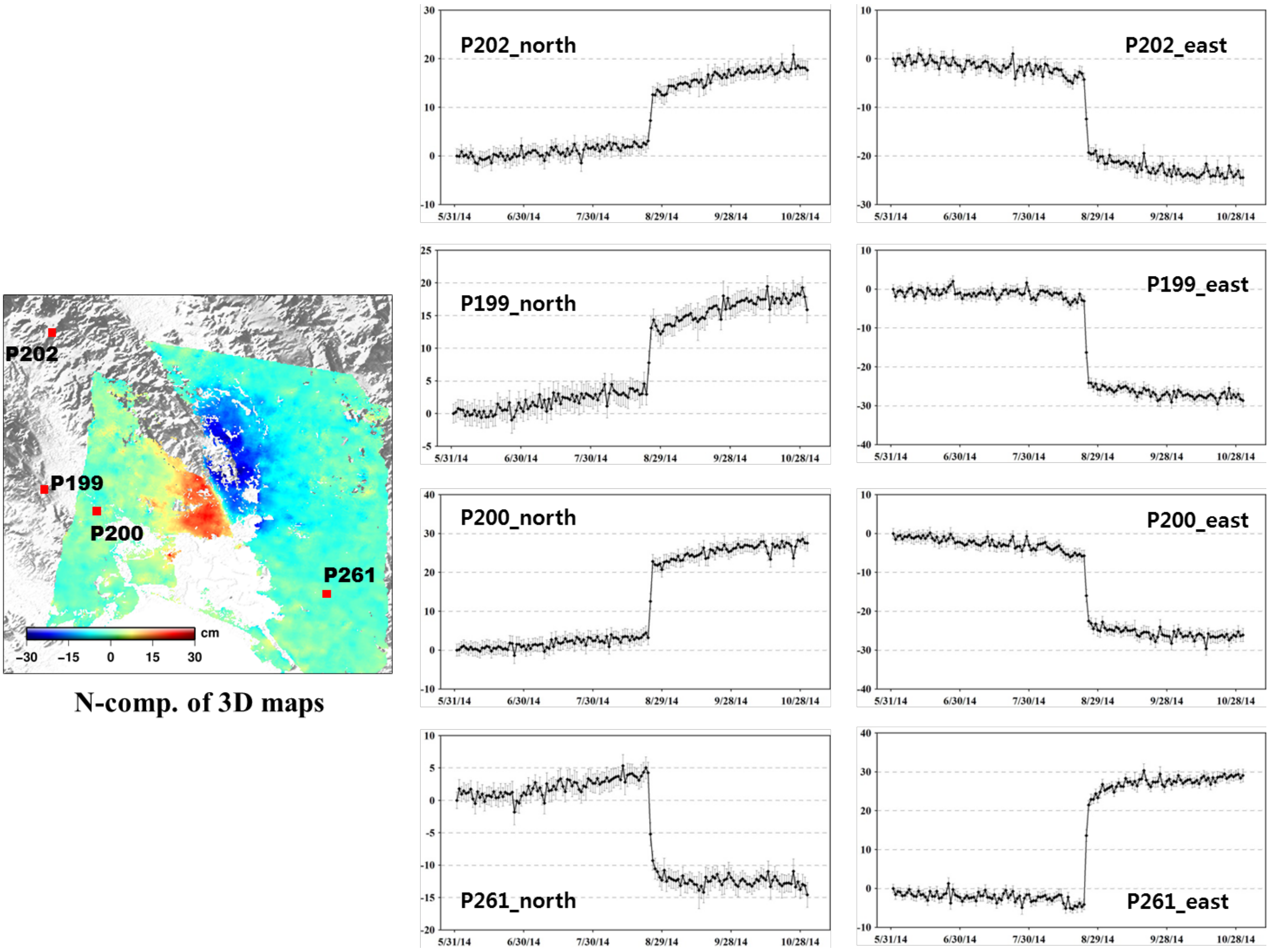

Fig. S3. Time-series GPS measurements for the stations which are located around the Napa fault. The map was generated by the GMT software 5.3.2 version (http://gmt.soest.hawaii.edu/projects/gmt/wiki/Download).

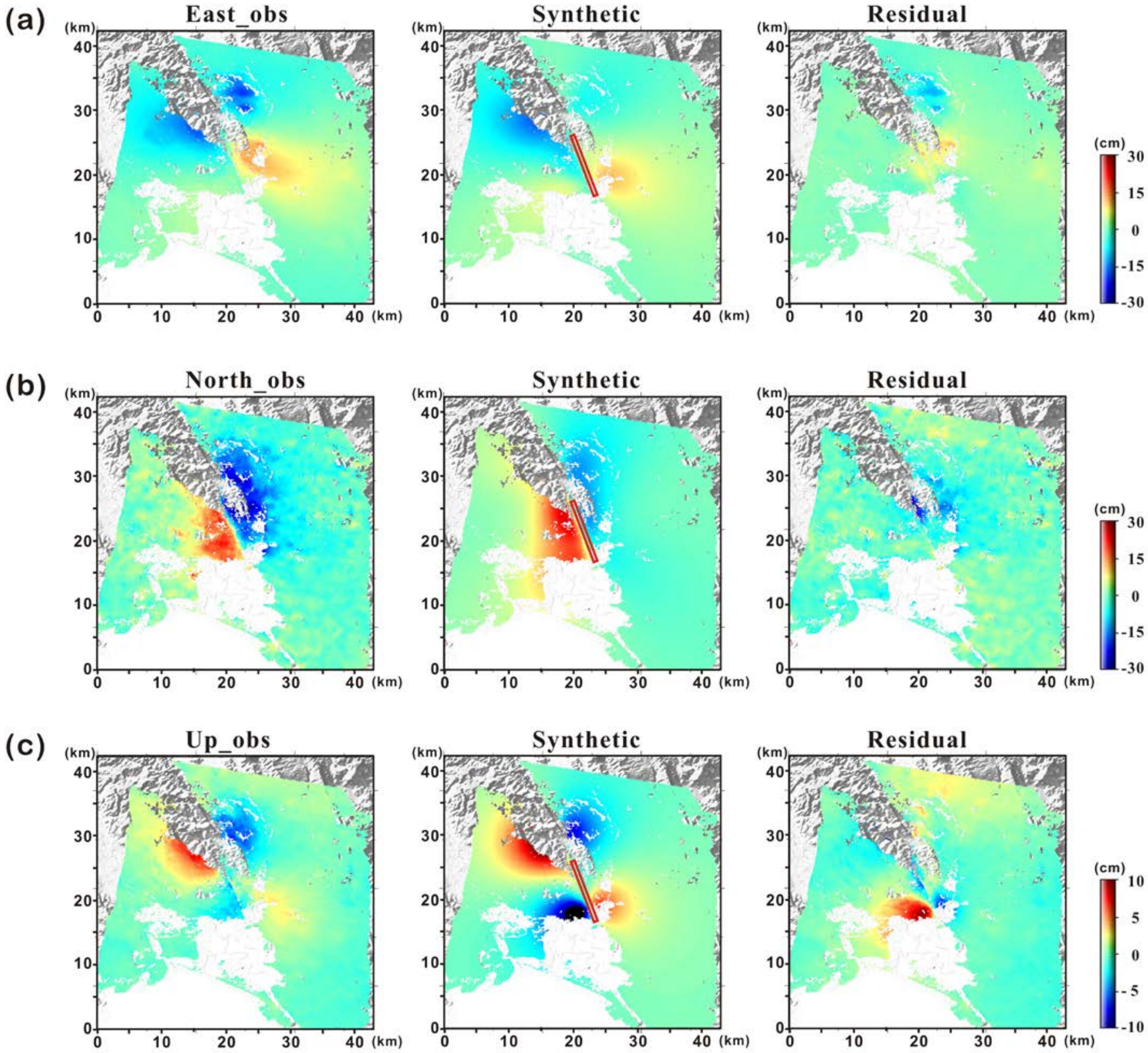

Fig. S4. Inversion results of 3D modeling assuming an east dipping fault. The maps were produced by using the GMT software 5.3.2 version (http://gmt.soest.hawaii.edu/projects/gmt/wiki/Download).
